# EJNMMI Physics - an editor's perspective

**DOI:** 10.1186/2197-7364-1-1

**Published:** 2014-05-01

**Authors:** Thomas Beyer, Igansi Carrio

**Affiliations:** Center for Medical Physics and Biomedical Engineering, General Hospital Vienna, Medical University of Vienna, Waehringer Guertel 18-20/4L, Vienna, 1090 Austria; Medical Imaging Cluster, Medical University of Vienna, Vienna, 1090 Austria; Nuclear Medicine Department, Hospital Sant Pau, Barcelona, 08026 Spain

**Keywords:** EJNMMI Physics, Open access, Journal

## Editorial

According to Wikipedia, an editorial is an ‘opinion piece written by the senior editorial staff’ and supposed ‘to reflect the opinion of the periodical’. This editorial is then somewhat different, as it opens the floor to a new journal, which has not yet any periodicity. However, this new journal deserves an introduction, and an editorial ever more.

*EJNMMI Physics* was launched in late 2013 at the Annual Meeting of the European Association of Nuclear Medicine in Lyon [1]. It pairs with the already existing *EJNMMI Research* journal, edited by Angelika Bischof-Delaloye. Both, the *Research* and the *Physics* journal, are open-access associates to *EJNMMI* that is edited by Ignasi Carrio and recently reported an impact factor of 5.113 [2].

*EJNMMI Physics* is far away from any impact factor, yet. We appreciate the appeal and the need of impact factors, and we are certain that together we can build this journal into a scientific platform for applied physics, which in due course can be awarded an impact factor. Our certainty stems from the fact that physics, while being an integral part of medicine, and nuclear medicine in particular, is underrepresented in the relevant medical journals. Therefore, *EJNMMI Physics* intends to support the presentation of physics and physics-related matters as an integral part of nuclear medicine in particular. It aims at providing a forum to scientifically minded people engaged and interested in nuclear medicine and associate imaging and therapeutic applications. As such, *EJNMMI Physics* complements its partner journal *EJNMMI Research* and supports *EJNMMI*, which focuses mainly on clinical perspectives of nuclear medicine.

*EJNMMI Physics* welcomes original materials and studies with a focus on applied physics, mathematics, as well as imaging system engineering and prototyping in nuclear medicine. This includes physics-driven approaches or algorithms supported by physics that foster early clinical adoption of nuclear medicine imaging and therapy regimens. More specifically, we introduce a number of manuscript categories to better reflect the current scope of physics-driven research in this particular field of medicine:

‘Original articles’ are manuscripts that describe unique scientific contributions, starting with a hypothesis, clearly defined materials and methods, presenting results and an unbiased discussion and conclusion. A special category of this type of manuscripts is ‘Short communications’ which describe unique scientific contributions based on an abbreviated study presenting first results of promising nature that appeals to the field of physics in nuclear medicine. Further to this, it is planned to introduce a category of ‘Young Investigator reports’ , which provides a platform for young investigators at an early stage in their career who seek to present findings from their scientific work as part of their diploma thesis and alike. It is planned that the EANM Physics and Dosimetry Committees select highlight contributions from this category to be awarded at the Annual Meeting of the EANMMI.

‘Artifact reports’ represent brief communications on frequent and infrequent artifacts of significant magnitude observed in routine applications of nuclear medicine imaging together with a narrative on the origin and solutions of these artifacts. This document could serve as guidance to picking up any such described image distortions and to take preventive measures or to apply illustrated correction measures.

Other contributions include standard formats, such as ‘opinion papers’ , ‘invited perspectives’ , ‘editorials’ , ‘letters to the editor’ , and ‘teaching files’ , of which the latter may be regarded as an expanded case review, a category that starts to disappear from other, more traditional journals, but which, we feel, provides relevant information in times when imaging is gaining ground and information is presented mainly visually.

Today, we are very happy to announce the first round of accepted manuscripts with this issue. This journal starts out with a series of invited perspectives on the role of Physics in Nuclear Medicine [3–6]. We have asked two key opinion leaders each, a nuclear medicine physician and a physicist, to share their view on the role of physics, either as a forward or as a backward look. This may be seem as an act of self-promotion, which is true, but it is also a very adequate reflection on the many contributions of physics and physics-minded people on a very important field of medical diagnosis and therapy. While this journal was launched only a few weeks ago, we are happy to report the receipt of the first original manuscripts for consideration for publication.

Finally, we like to thank those people who volunteer to serve on the Editorial Board of *EJNMMI Physics*, making this a respectable arena for physicists and friends of physics: Soren Baarsgaard Hansen (Aarhus University Hospital, Denmark), Dale Bailey (Royal North Shore Hospital, Australia), Wolfgang Birkfellner (Medical University of Vienna, Austria), Ronald Boellaard (VU University Medical Centre, Netherlands), Ciprian Catana (Massachusetts General Hospital, Harvard Medical School, USA), Alberto del Guerra (University of Pisa, Italy), Gaspar Delso (University Hospital Zurich, Switzerland), Glenn Flux (Institute of Cancer Research, UK), Dietmar Georg (Medical University of Vienna, Austria), Gerhard Glatting (Medical Faculty Mannheim, University of Heidelberg, Germany), Hans Herzog (Niederzier, Germany), Brian Hutton (University College London, UK), Marc Kachelriess (German Cancer Research Center, Germany), Claudia Kuntner (Austrian Institute of Technology GmbH, Austria), George Loudos (Technological Educational Institute of Athens, Greece), Stan Majewski (West Virginia University, USA), Osama Mawlawi (MD Anderson Cancer Center, USA), Ewald Moser (Medical University of Vienna, Austria), Otto Muzik (Wayne State University Medical School, USA), Uwe Pietrzyk (Forschungszentrum Jülich, Germany), Harald Quick (University of Duisburg-Essen, Germany), Bernhard Sattler (University Hospital Leipzig, Germany), Piotr Slomka (Cedars-Sinai Medical Center, USA), Joerg van den Hoff (Helmholtz-Center, Dresden, Germany), Dimitris Visvikis (University of Brest, France), and Charles Watson (Siemens Molecular Imaging, USA). Without a doubt, their scope of expertise covers a wide range of Physics in the realms of applications in medicine, and nuclear medicine in particular.

And now, the floor is open to all who like to see Physics grow in within Nuclear Medicine and to get applied in real-world scenarios.
